# Light and Hydrogels: A New Generation of Antimicrobial Materials

**DOI:** 10.3390/ma14040787

**Published:** 2021-02-07

**Authors:** Lucie Pierau, Davy-Louis Versace

**Affiliations:** Institut de Chimie et des Matériaux Paris-Est (ICMPE) UMR-CNRS UPEC, 2-8 rue Henri Dunant, 94320 Thiais, France; lucie.pierau@glvt-cnrs.fr

**Keywords:** antibacterial hydrogels, photochemistry, free-radical photopolymerization, photodynamic inactivation of bacteria

## Abstract

Nosocomial diseases are becoming a scourge in hospitals worldwide, and new multidrug-resistant microorganisms are appearing at the forefront, significantly increasing the number of deaths. Innovative solutions must emerge to prevent the imminent health crisis risk, and antibacterial hydrogels are one of them. In addition to this, for the past ten years, photochemistry has become an appealing green process attracting continuous attention from scientists in the scope of sustainable development, as it exhibits many advantages over other methods used in polymer chemistry. Therefore, the combination of antimicrobial hydrogels and light has become a matter of course to design innovative antimicrobial materials. In the present review, we focus on the use of photochemistry to highlight two categories of hydrogels: (a) antibacterial hydrogels synthesized via a free-radical photochemical crosslinking process and (b) chemical hydrogels with light-triggered antibacterial properties. Numerous examples of these new types of hydrogels are described, and some notions of photochemistry are introduced.

## 1. Introduction

For the past ten years, the use of antibiotics has led to the emergence of multidrug-resistant microorganisms responsible for the exponential increase of nosocomial diseases in hospitals, also known as healthcare-associated infections (HAIs). As an appalling consequence, several million patients have contracted nosocomial infections during their stay at the hospital annually. It has been estimated that among the nearly 1.7 million hospitalized patients annually acquiring HAIs while receiving treatment for different health issues in the US, 1 patient in 17 dies due to HAIs [1]. In advanced and emerging countries, seven and ten hospitalized patients out of 100, respectively, acquire HAIs. As well as these alarming statistics, the US government spent at least $10 billion annually to struggle against HAIs, according to the Office for National Statistics and these additional costs are directly charged to the hospital’s budget [1,2,3]. The proliferation of microorganisms should be urgently restricted because the estimated mortality rate is expected to become higher than that of cancers, inexorably raising the healthcare costs and social security debt [4,5].

To address this matter, the research and development of antibacterial materials sky-rocketed over the last couple of years due to the imminent health crisis risk. Challenged by the emergence of new pathogenic microorganisms, researchers have developed innovative advanced strategies to eradicate resistant bacteria responsible for HAIs [6,7,8,9,10,11,12,13,14,15,16], and many investigations [17,18,19,20,21,22] have been thus published. Antimicrobial materials could be classified as (i) biocides materials or “active” materials when they provoke bacterial death either by contact or from the release of biocidal agents and (ii) anti-fouling or “passive” materials when adhesion and/or proliferation of bacteria onto their surface are prevented without leading to death. Among the most relevant “active strategies”, we can cite the use of reactive oxygen species [7,9], polymers with quaternary ammonium functional groups [23,24], natural peptides [25,26], phospho- and sulfo-derived polymers [27,28], phenol and benzoic acid derived polymers [15], polyelectrolyte multilayers [29], or metal-based inorganic antimicrobial compounds such as zinc oxide, TiO_2_, or metal nanoparticles [10,12,30,31]. On the contrary, the use of PEG-containing or fluorinated-based materials is well-known to hinder the adhesion or proliferation of bacteria according to a “passive strategy” [32,33].

Among all the most efficient described strategies, hydrogels have been considered as excellent candidates for effective antibacterial applications. Hydrogels are a class of soft polymeric materials that have been playing a significant role in numerous fields since they first came to light in the mid-20th century [34,35,36]. Briefly, hydrogels are a hydrophilic three-dimensionally crosslinked polymeric network. They are obtained through gelation, a process that occurs when water-soluble monomeric units and polymeric chains physically or chemically crosslink together to form a non-soluble 3D porous structure [34,35,36,37]. One of their main characteristics is their ability to retain a substantial amount of water in their swollen state because of hydrophilic entities such as hydroxyl, amino, ether or carboxylic groups in their polymeric chains while being insoluble in water. Their preparation typically requires three components: monomers, oligomers or polymers, an initiator and a crosslinker [34].

Hydrogels have been exhaustively described in the literature by several outstanding reviews [34,35,36,37,38]. They are broadly classified into many subtypes based on:

*Origin*: hydrogels can be made from natural, semi-synthetic or synthetic substrates;

*Durability*: whether hydrogels are durable or biodegradable;

*Composition of the polymers*: homo-polymeric or copolymeric hydrogels;

*Response to external stimuli*: physical stimuli such as electric field, magnetic field, temperature or light, and chemical stimuli such as pH conditions, solvent composition, or ionic strength;

*Physical state*: hydrogels can be found in the solid, semi-solid or liquid state;

*Structural details*: physical configurations, amorphous, semi-crystalline or crystalline systems;

*Charges on the polymeric configurations*: nonionic or neutral hydrogels, ionic—anionic or cationic—hydrogels, ampholytic hydrogels or zwitterionic hydrogels;

*Gelation type*: whether hydrogels are physically or chemically crosslinked.

Owing to considerable variation of compositions, extensive scientific research on the subject and advances in synthetic chemistry, hydrogels can be tailored to meet specific needs depending on their field of application. Their tunable mechanical and rheological properties, their sensitivity towards external stimuli and their ability to provide a moist environment and high levels of biocompatibility make them particularly interesting materials in the pharmaceutical and biomedical worlds [34,37,38]. Their extensive usage ranges from tissue engineering [39,40], wound dressing [37,41,42], antibacterial coatings [43,44,45], contact lenses [46,47], drug delivery systems [48,49], super-absorbents [35,50,51], biomedical implants [52] as well as a great number of other therapeutic applications [34,53], to miscellaneous applications such as biosensors [54], microelectromechanical devices [35], electrical conduction [55], energy storage devices [56,57], purification of water and decontamination of organic waste [50,56,58,59], food and agricultural applications [60].

Nowadays, hydrogels clearly appear as alternative materials for antibacterial applications. However, despite the existence of two types of hydrogels, i.e., physical hydrogels, which crosslinking between polymer chains is due to non-covalent interactions (hydrogen bonding, ionic forces, Van der Waals interactions, polyelectrolyte complexation, or hydrophobic forces), and chemical hydrogels, only the chemical ones will be highlighted in this study. Indeed, chemical hydrogels involve strong covalent bonding between the polymer chains, and the resulting hydrogels show excellent thermal, mechanical and chemical properties while maintaining the 3D hydrogel network structure in a fully swollen state.

The antibacterial properties of hydrogels can be tuned either by simply modifying their intrinsic properties such as hydrophilicity, porosity or by successfully selecting monomers and crosslinkers. According to the classification of hydrogels matrices and the nature of the antimicrobial agents, two distinguished categories of antibacterial hydrogels can finally be outlined: hydrogels that have intrinsic antibacterial properties when formulated with cationic polymers or antimicrobial peptides and hydrogels that exhibit antibacterial activity when loaded with antibacterial agents, such as antibiotics or metal nanoparticles (NPs) [38,61]. In the latter case, the bactericidal effects occur and are often only effective upon the slow leaching of the antibacterial agent into the surrounding environment [44]. This phenomenon constitutes one of several drawbacks of materials loaded with leaching antibacterial agents, inducing environment contamination, and generating short periods of bactericidal action due to rapid leaching at the beginning of use. In order to address this matter, several approaches have been investigated to either make non-leaching antibacterial agent-loaded materials [48] or material surfaces with intrinsic permanent biocidal activity on contact [41,61,62]. Some of these research studies, associated with either one of these strategies and sometimes even both [44], are further described in this review. Finally, reactive oxygen species (ROS) such as oxygen, peroxide and hydroxide radicals or singlet oxygen can also lead to the death of bacteria by oxidizing their cellular constituents and by denaturing bacterial DNA, RNA and proteins [63]. This type of antibacterial action will also be further explained in this work.

**Scheme 1:**
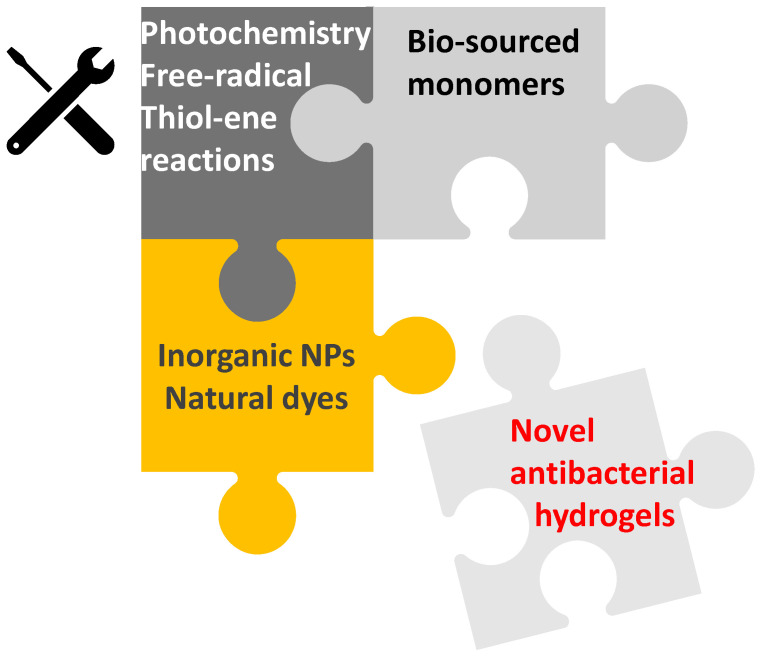
Synthesis of novel photoinduced and antibacterial hydrogels.

Interestingly, many of these investigations concern the design of antibacterial hydrogels [64] via thermal free-radical polymerization, and a few have exploited the striking advantages of photochemistry over the thermal process [62,65,66,67]. Yet, photochemistry is now well recognized as a green method and has attracted great attention because it offers many advantages over thermal processes [68,69,70]. To the best of our knowledge, and despite the advantages of the green photochemistry process [65], no review has been made on the synthesis of antibacterial hydrogels by photochemistry technology yet. This encouraged us to highlight and summarize how some recent studies within this framework investigated and benefited from visible-light properties for the synthesis and application of such hydrogels (Scheme 1). Specifically, we focus on the following two categories: (a) antibacterial hydrogels obtained via a free-radical photochemical crosslinking process and (b) hydrogels with light-triggered antibacterial properties. Some notions of photochemistry have also been introduced for a better comprehension of the review.

## 2. Light as a Promising Source of Energy

### 2.1. Why Use Photochemistry?

Most of the research that has been made on hydrogels over the year focused on their synthesis using heat as a source of energy. It is only until recently that the synthesis of antibacterial hydrogels by photochemistry has been described [61,62,69].

Photochemistry is a very appealing green process attracting continuous attention from scientists in the scope of sustainable development, as it exhibits many advantages over other methods used in polymer chemistry, as exhaustively described by brilliant reviews [69,71,72].

Among the benefits brought by photochemical processes [68,70,71], rapid curing is the most notable one. Processes that do not readily occur or that are unable to occur at ambient temperature on a reasonable timescale because they lack sufficient amounts of energy to overcome the energetic barrier can rapidly occur upon exposure to the appropriate light source. Upon heating, polymerizing systems can easily take hours to cure, whereas photoinitiated systems occur under a few seconds or minutes.

Second, as one of the primary driving forces for the reaction to occur is the absorption of a photon by a photosensitive compound, such photoinitiated processes can generally be temporally controlled by simply switching on and off the light–source. This method also requires very little energy as it can be done at very low light intensities. In addition, the development of new performing light-emitting diodes (LEDs) and the wide range in wavelengths and intensities of light sources available are supplementary assets contributing to make photochemistry a very attractive field.

Initiating a reactive system by absorbing photons also enables such photoinitiated processes to prevent the use of harsh environmental conditions, such as elevated temperature, the use of organic solvents or toxic components. Not only do these benefits render photochemistry more likely to be compatible with biomolecules [62], but it also avoids the release of volatile organic compounds. This also implies low levels of waste and low costs of investment.

Finally, another advantage of photochemistry is the spatial control of the considered reaction. Using masking or patterned illumination thanks to light-sources coupled to mirrors, it is possible to focus light on a particular surface or within a specific volume to achieve two-dimensional and three-dimensional spatial control, respectively.

### 2.2. Which Photoinitiating Systems Used for Hydrogel Synthesis?

While photochemistry brings great advantages to polymer science and chemistry in general, another one of its assets consists of the broad range of generic reaction types that can be photo-initiated for the synthesis of polymers [69]. The description, applications, benefits and restrictions of these photo-initiated reaction types have already been exhaustively outlined in compelling reviews [69,71,72].

Broadly, the two most common types of photopolymerization are free-radical photopolymerization (FRP) and cationic photopolymerization (CP) [71,72]; however, FRP is the exclusive process used in hydrogel synthesis. Only FRP will be evoked in the following review, and special interest will be given to the thiol-ene process as well.

Basically, this formulation consists of: (i) a monomer/oligomer with photoactivable groups, (ii) a photoinitiator (PI) or a photoinitiating system (PIS), which could initiate the polymerization under light exposure and (iii) different additives (plasticizers, fillers, pigments) to adjust and tailor the properties of the formulation. The mechanisms involved in the photopolymerization reactions are described below. The discussion will focus here on the most common photopolymerization reaction for hydrogel synthesis related to FRP and thiol-ene processes. However, some essential notions of photochemistry will first be addressed.

According to the type of PIS chosen, different mechanisms can be expected, i.e., a Norrish type I mechanism or a bimolecular mechanism called Norrish type II. Novel Norrish type I and type II photocleavable systems, acting in the visible or infrared (IR) range, have been thus synthesized; their structures and photochemical properties have also been extensively reviewed [68,70,71].

#### 2.2.1. Norrish Type I Mechanism

**Figure 1:**
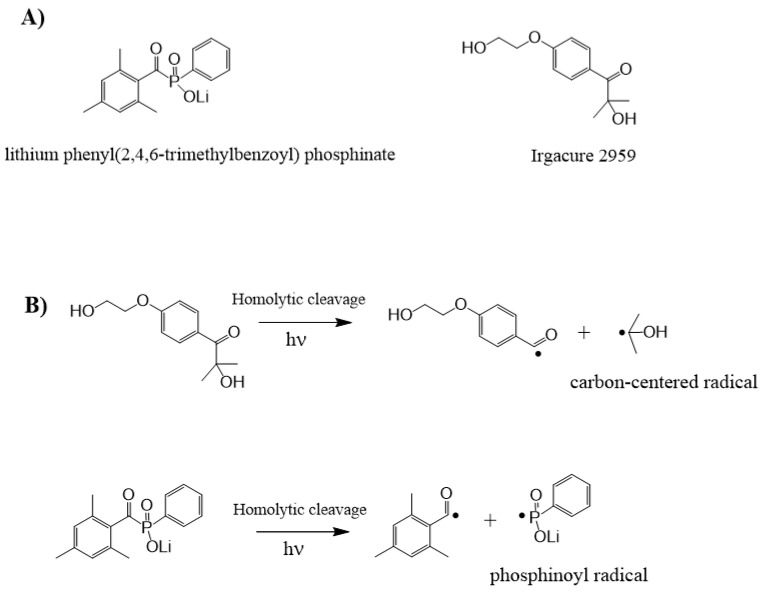
(**A**) Examples of Norrish type I photo-initiators and (**B**) mechanism of the homolytic cleavage of Norrish type I photo-initiators under light irradiation.

Under light irradiation, if a photoinitiator (PI) can undergo a homolytic cleavage generating two active radical species, the compound of this type is known as a Norrish type I PI. This reaction is only possible if the dissociation energy of the PI in its ground state is lower than the excitation energy. Most of the type I PIs (aryl ketones) are composed of aromatic moieties containing carbonyl groups. For instance, 2-hydroxy-1-[4-(hydroxyethoxy)phenyl]-2-methyl-1-propanone (Irgacure 2959, Figure 1A) or lithium phenyl(2,4,6-trimethylbenzoyl) phosphinate (Figure 1A) are well-established PIs used in the synthesis of hydrogels under light irradiation with weak toxicity over a range of mammalian species [73]. After the photoinduced α-cleavage reaction (homolytic cleavage) between an aromatic and a carbonyl group, two carbon-centered (or phosphinoyl) radicals are produced, and their reactivity depends on the attached functional side-groups (Figure 1B). Generally, only the generated alkyl (and phosphinoyl) radicals are reactive toward the vinyl groups and may initiate FRP.

#### 2.2.2. Norrish Type II Mechanism

**Figure 2:**
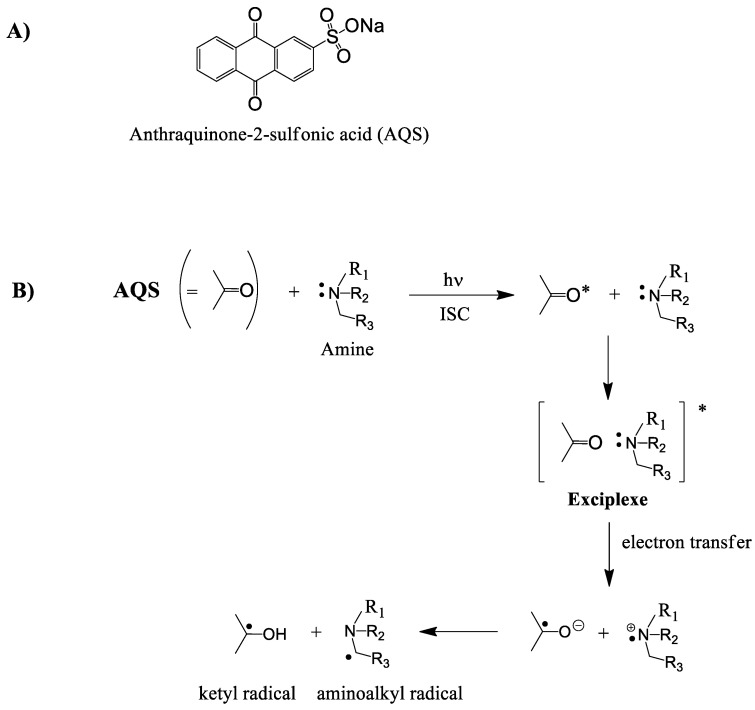
(**A**) Example of a type II photoinitiator and (**B**) mechanistic approach with anthraquinone-2-sulfonic acid (AQS) under light irraditation.

The Norrish type II reactions can occur according to two processes: the first one is a hydrogen abstraction reaction (a bimolecular reaction) between a PI and a hydrogen donor molecule (AH), forming thus two radicals; the second one concerns the electron/proton transfer reaction between a PI and an electron-donating molecule with labile hydrogen (DH) via a charge transfer complex (CTC or exciplex). The resulting A• or D• radicals can initiate FRP. Sautrot et al. have recently used anthraquinone-2-sulfonic acid (AQS) coupled with *N*-methyldiethanol amine (MDEA) as an H-donor molecule [61] (Figure 2).

#### 2.2.3. Free-Radical Photopolymerization Process

**Figure 3:**
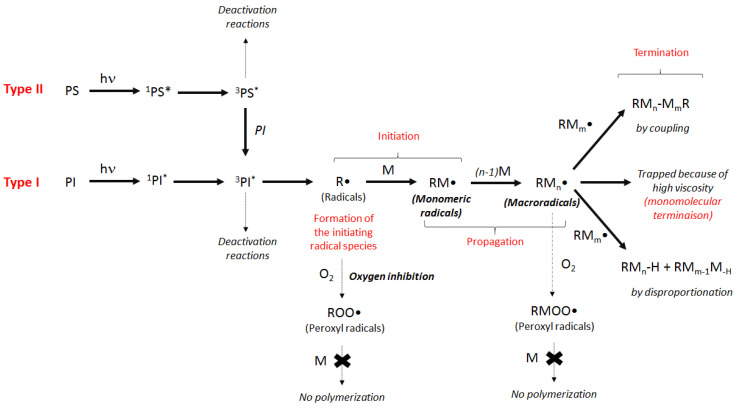
Overall mechanism of free-radical photopolymerization (FRP) with and without oxygen under light activation.

After considering the formation of radical active species, the overall simplified mechanism of FRP with and without oxygen is represented in Figure 3. During the first stage of the process, the initiating radical species (R•) are generated through mono or bimolecular reactions as previously described. R• reacts, for example, with (meth)acrylate monomers and leads to the monomeric radical (RM•). Subsequently, the propagation step occurs when monomers additionally react with the growing macroradical. Finally, the termination reaction can take place according to the mono or bimolecular process. The latter occurs through recombination of macroradicals (coupling) or by disproportionation from a H-abstraction reaction. The monomolecular termination is preferentially observed when the formulation becomes highly viscous so that the diffusion of the radical species is prevented and cannot combine together.

Despite the use of FRP in many syntheses, oxygen inhibition stays a serious concern. Indeed, oxygen reacts with monomeric or macroradicals to form peroxyl radicals (ROO•) whose slow down or inhibit the polymerization. To address this issue, numerous new systems have been designed, and new co-initiators have been introduced in the photosensitive formulations, such as silanes, *N*-vinylcarbazole, halogen or bore-derived compounds [68,71].

#### 2.2.4. Initiation by the Thiol-Ene Process 

**Figure 4:**
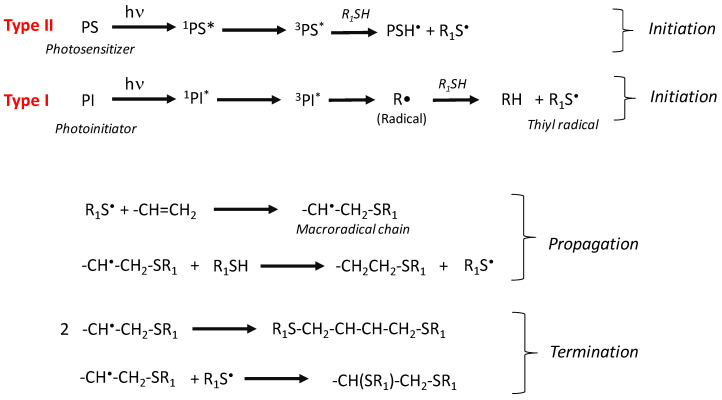
Overall mechanism related to the thiol-ene process.

Thiol-ene polymerization was first evidenced by Sharpless and is dedicated to the addition of thiyl radicals to the double bond of multifunctional olefins (acrylate, methacrylate, allyl or vinyl). Thiol-ene polymerization is characterized by striking features such as no oxygen inhibition, fast reaction process and synthesis of highly crosslinked and dense networks with high T_g_, high rubbery modulus, high adhesion and reduced shrinkage [74,75,76,77]. Many syntheses of hydrogels have been devoted to the thiol-ene polymerizations, and this technique has been recently revitalized through many applications [78,79,80]. The so-called thiol-ene mechanism is described in Figure 4: (i) Norrish type I or type II PI in combination with thiols are appropriate to initiate the thiol-ene process according to an H-abstraction reaction, generating thiyl radicals, (ii) propagation mechanism with the addition of thiyl radicals to double bond and regeneration of thiyl radicals and (iii) bimolecular termination reaction between two macroradical chains or between a thiyl radical and a macroradical chain.

## 3. Photochemistry and Antibacterial Hydrogels: Biological Applications

Two topics can be underlined when talking about photochemical processes and antibacterial hydrogels: on one hand, there are photoinduced antibacterial hydrogels, i.e., hydrogels whose synthetic process uses photochemistry as the triggering source of energy for crosslinking; on the other hand, there are hydrogels whose bactericidal effects are due to reactive oxygen species generated from light activation.

### 3.1. Photoinduced Antibacterial Hydrogels

The antibacterial part of such photoinduced hydrogels comes from intrinsic bactericidal properties when formulated with cationic polymers or antimicrobial peptides (organic photoinduced antibacterial hydrogels), or if the considered hydrogel is loaded with antibacterial agents, such as metal NPs or metal–organic frameworks (MOFs), for instance (hybrid organic–inorganic antibacterial hydrogels).

So far, most of the photoinduced antibacterial hydrogels have been investigated within the scope of biomedical purposes, mainly regarding HAIs. To address this matter, antibacterial hydrogels obtained using photochemical processes have recently gained increasing interest owing to the many advantages brought by photochemistry [69,71,72]. The following studies investigated the photoinduced synthesis of polymeric networks exhibiting antibacterial properties for in situ biomedical applications such as wound dressings and injectable antibacterial hydrogels.

#### 3.1.1. Organic Photoinduced Antibacterial Hydrogels

**Figure 5:**
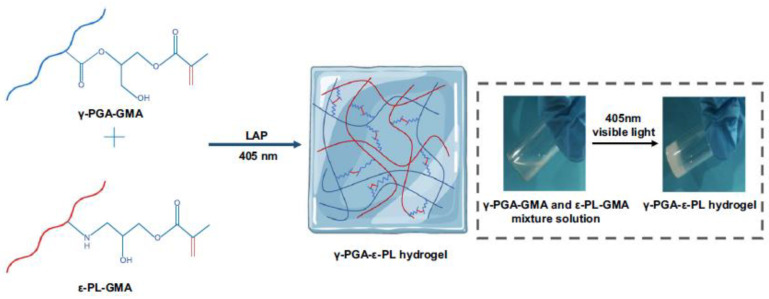
Schematic representation of light-polymerized hydrogel based on γ-poly(glutamic acid) (γ-PGA)–ε-poly-L-lysine (ε-PL) hydrogel synthesis. Reprinted with permission. Copyright 2021, NPG Asia Materials [41].

Thanks to rapid curing, light enables the investigation of photoinduced antibacterial hydrogels for in-situ injectable wound dressings. It is also particularly interesting for hydrogels synthesized using natural polypeptides and biomolecules considering the mild reactional conditions. Sun et al. reported the synthesis of an in situ injectable light-polymerized hydrogel based on γ-poly(glutamic acid) (γ-PGA), a poly-amino acid obtained via microbial fermentation, and ε-poly-L-lysine (ε-PL), a water-soluble, biocompatible and antibacterial polypeptide [41]. They mixed solutions of methacrylated (γ-PGA) and (ε-PL) with a visible-light initiator, lithium phenyl(2,4,6-trimethylbenzoyl) phosphinate (LAP), to obtain upon irradiation by visible light (405 nm) at room temperature a biocompatible and biodegradable broad-spectrum antibacterial hydrogel (Figure 5). It was tested in vitro against *Staphylococcus aureus* (*S. aureus*) and *Escherichia coli* (*E. coli*), model microorganisms for testing bactericidal properties, and in vivo to treat models of subcutaneous infection and damaged skin on rats. In parallel, they also assessed its mechanical properties as well as its biocompatibility.

Their results showed a fast gelation step, 6 to 10 s, depending on the proportion of methacrylated ε-PL, indicating promising injectable applications. The γ-PGA–ε-PL hydrogel exhibited homogenous pore sizes enabling nutrient exchange and cell migration. Additionally, high levels of antibacterial activities were obtained as the outcomes showed that for some samples, over 99% of *S. aureus* and *E. coli* strains were killed. Cytotoxicity and wound closure assessments indicated excellent hydrogel biocompatibility as well as a good potential for wound healing applications. Overall, they obtained a visible-light-induced, homogeneous and stable crosslinked hydrogel, which featured a high level of biocompatibility, inhibited bacterial activity, showed killing capacity against *S. aureus* and *E. coli* through cell membrane disruption and promoted wound repair.

Another study in the framework of HAIs using naturally occurring intrinsically antibacterial polymers was conducted by Sautrot-Ba et al. [62]. They investigated chitosan (CS), a natural polycationic linear polysaccharide frequently involved in the synthesis of hydrogels, and more particularly for wound dressing materials and drug-releasing systems, because of its biocompatibility, biodegradability, non-toxicity, relative abundance in nature and natural antibacterial activity (fungistatic and bacteriostatic activities).

After chemical modification, primary amino groups located alongside the polymeric chain of chitosan can be turned into quaternary ammonium (QA) functional groups [81]. QA functional groups feature great antibacterial activity due to their positive charge, which is more likely to interact with the negatively charged cell wall of the outer membrane of Gram-positive and Gram-negative bacteria, causing pore formation in the cell membrane, severe cell constituent leakage and inducing bacterial death via membrane disruption [61,62]. Chitosan is an interesting platform in the framework of antibacterial hydrogels as it can be modified in photopolymerizable chitosan derivatives. These derivatives allow the introduction of chemical crosslinks in polysaccharide hydrogels and/or the creation of new hybrid materials via combination with synthetic entities [62].

**Figure 6:**
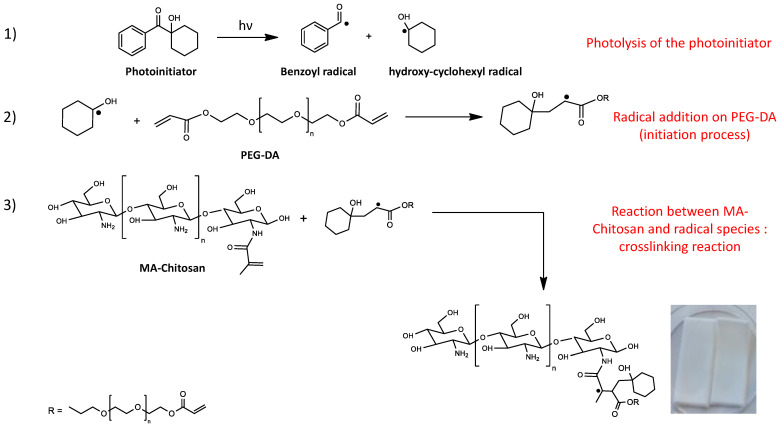
Schematic representation of the free-radical photoinduced crosslinking between poly(ethylene glycol) dimethacrylate (PEG-DA) and methacrylated chitosan (MA-Chitosan) and optical image of the resulting hydrogel. (**1**) Photolysis of 1-hydroxy-cyclohexyl-phenyl-ketone, (**2**) radical addition on PEG-DA (initiation process) and (**3**) Reaction between MA-Chitosan and radical species (crosslinking reaction) Reprinted with permission. Copyright 2019, Royal Society of Chemistry [62].

In the scope of their study, Sautrot-Ba et al. synthesized in aqueous conditions a hybrid antibacterial hydrogel composed of a chitosan-based polymer modified with methacrylic acid, and poly(ethylene glycol) dimethacrylate (PEG-DA) in the presence of a photoinitiator, 1-hydroxy-cyclohexyl-phenyl-ketone, combining thus natural anti-fouling properties of PEG and the natural antibacterial properties of chitosan (Figure 6). They irradiated their mixtures for 100 s under a Hg–Xe lamp (250 W, 250–700 nm range) and used photo-rheology and real-time Fourier-transform infrared spectroscopy to describe in detail the photochemical behavior of such a system upon light irradiation. For the first time, they also studied the antibacterial activity of their hybrid hydrogels in the long-term, performing bacterial adhesion experiments after 2 and 6 h and up to 6 months after having obtained their hydrogels against both Gram-positive *S. aureus* and Gram-negative *E. coli* bacteria.

According to their results, it seems that incorporating chitosan within the photo-crosslinked structure slows down the photopolymerization, and the same goes when they decrease the amount of PEG-DA. However, it does so without impacting their final conversion rates, ranging from 90 to 100%. Their study also suggests that the introduction of chitosan within the polymeric network decreases the crosslinking density, thus increasing the swelling ratio of the resulting material. However, it also involves a decrease of the water content at equilibrium due to the more hydrophobic character of the chitosan chain compared to PEG. On the other hand, if a decrease of PEG content within the polymeric network reveals the same trend in terms of crosslinking density and swelling capacity, in that case, it implies a larger water content at the equilibrium of the hydrogel in its swollen state. They also observed that the methyl acrylamide chitosan-PEG derivative bridges forming during the photochemical process occur at the expense of the hydrogel thermal and mechanical stability. Concerning the antibacterial properties of their material post-synthesis, their data showed that the chitosan-containing gels featured an anti-fouling capacity against both tested bacteria as well as bactericidal effects against *E. coli*. These biocidal effects were less significant against *S. aureus*, which they explained by the difference in thickness of the peptidoglycan layer between bacteria and the chitosan accessibility to the bacteria depending on its repartition within the PEG network. After six months of storage in a refrigerator at +2 °C, the results of their second antibacterial experiments showed that the hydrogels suffered no loss of material during storage, indicating good chemical integrity, although the hydrogels probably underwent some structure reorganization, including chitosan chains accessibility on the hydrogel surface during that time period since they observed a slight bactericidal efficiency loss.

**Figure 7:**
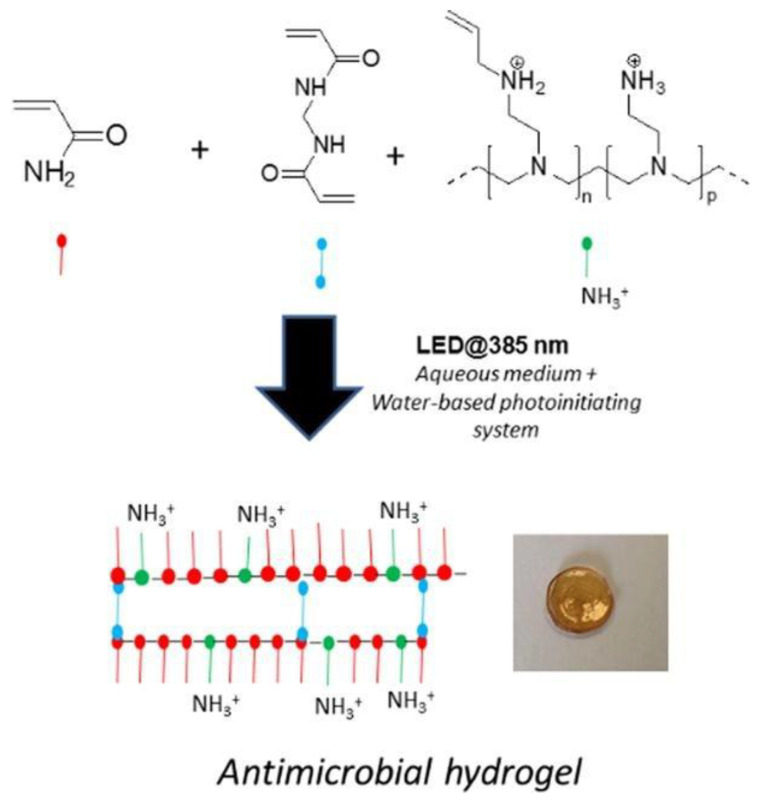
Schematic illustration of the photoinduced crosslinking between acrylamide, bisacrylamide and allylated poly(ethyleneimine) forming an antibacterial hydrogel. Reprinted with permission. Copyright 2020, Elsevier [61].

Recently, the same research group described the synthesis of antibacterial gels combining acrylamide- and allylated poly(ethyleneimine) (A-PEI)-based monomers using radical photopolymerization in aqueous conditions under air. They described a water-based photoinitiating system composed of anthraquinone-2-sulfonic acid (AQS), a photoinitiator showing strong light absorption in near-UV, and *N*-methyldiethanol amine (MDEA), a co-initiator [61]. The use of allylated PEI here is interesting as it is both used as a photo-crosslinker and as an antibacterial agent with the presence of quaternary ammonium (QA) functional groups. These QA functional groups are due to the presence of vinyl functional groups on the PEI backbone and are obtained by nucleophilic substitution reactions between the primary amino functional groups of PEI and allyl bromide. They mixed bisacrylamide, acrylamide, AQS and MDEA with their modified PEI in water under UV irradiation (385 nm) for 20 min (Figure 7). The mechanical and rheological properties, as well as the in vitro biocide activities against *S. aureus* and *E. coli* of the resulting gels were then tested. Their investigation showed that the addition of A-PEI in their photoreactive system accelerated the crosslinking reaction and improved the flexibility of the resulting network, thus increasing the swelling ratio (a flexible network physically allows water to diffuse within the polymeric structure). A good swelling enables bacteria to come in contact with the QA functional groups of the three-dimensional polymeric structure, increasing the odds of bacterial death. As for the antibacterial tests, not only the A-PEI-based gel they obtained showed antibacterial activity, but it also featured anti-adherence abilities, making this photoinduced crosslinked hydrogel both a passive and active material.

#### 3.1.2. Hybrid Organic–Inorganic Photoinduced Antibacterial Hydrogels

On the topic of PEG-based photoinduced antibacterial hydrogels, Gwon et al. described the synthesis of the antibacterial metal–organic framework (MOF)-embedded hydrogel using PEG-DA and 4-arm thiolated PEG (4-arm PEG-SH) via thiol-ene photopolymerization upon UV irradiation [48]. MOFs have recently gained interest from the scientific community as an efficient trapping structure for transition metal ions showing excellent antibacterial activities that could not be used as such due to excessive metal ion leaching, inducing cytotoxicity to host tissues as well as to bacteria [82]. Via the coordination of bioactive metal ions to organic bridging ligands, scientists hope to efficiently trap metallic entities within MOFs, avoiding thus metal ion leaching while ensuring effective antibacterial activity. Lipids present in bacterial membranes can easily be oxidized by active metal sites of MOF crystal surfaces interacting with the membrane, inducing then disruption and bacterial deactivation. Nevertheless, such structures still release significant amount of bioactive metal ions into the media through the decomposition of metal–ligand bonds, causing harm to host tissues [48].

Through surface coating, Gwon et al. hoped to increase the stability of metal–ligand bonds within MOFs to prevent excess metal ion release in MOF-based drug delivery systems. After having synthesized copper, cobalt and zinc MOFs, they added UV photoinitiator Irgacure 2959 to a polymeric aqueous solution composed of PEG-DA and 4-arm PEG-SH in a 1:1 molar ratio. Each MOF was then added to the prepared polymer precursor solutions and photo-crosslinked under UV light (365 cm) for 5 min to generate crosslinked bioactive MOF-based hydrogels. The mechanical properties, the cytotoxicity as well as the antibacterial activity against *E. coli* and *S. aureus* of the resulting materials were then investigated.

**Figure 8:**
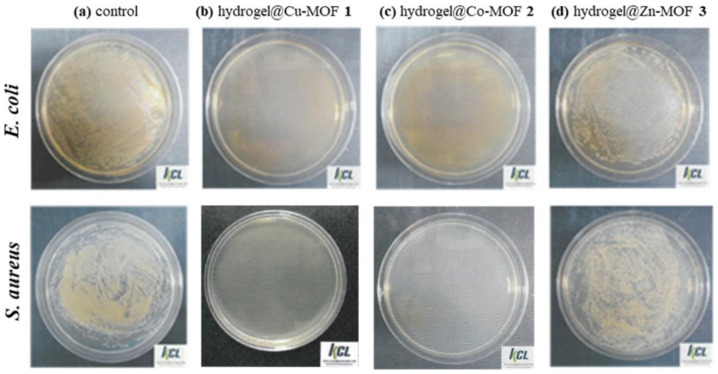
Assessment of antibacterial effects after 24 h of incubation by colony-forming assay on different metal–organic frameworks (MOFs)-encapScheme 105. cfu. mL^−1^). Reprinted with permission from ACS Appl. Mater. Interfaces 2020, 12, 20234−20242. Copyright 2020, American Chemical Society [48].

**Figure 9:**
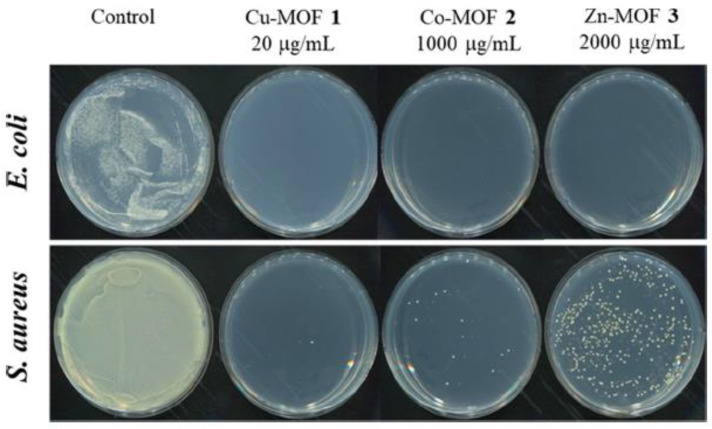
Assessment of antibacterial effects after 24 h of incubation by colony-forming assay on agar plates treated with MOFs (*E. coli* and *S. aureus* at 10^8^ and 10^7^ cfu. mL^−1,^ respectively). Reprinted with permission from ACS Appl. Mater. Interfaces 2020, 12, 20234−20242. Copyright 2020, American Chemical Society [48].

Their analysis confirmed the homogeneous encapsulation of MOFs within the hydrogel networks during the photo-crosslinking process, and they reported the obtention of a biocompatible, stable, bioactive Cu-based MOF-embedded hydrogel with high antibacterial activity against both *E. coli* and *S. aureus*. In their analysis, they pointed out that the central metal and structure of MOFs encapsulated in the polymers influenced the PEG-based hydrogel structures. Indeed, Cu-MOF featured stable chemical bonding with the PEG network as opposed to Zn-MOF and Co-MOF, which showed relatively unstable chemical bonding with their PEG structures. In terms of antibacterial activity, the Cu-MOF-encapsulated hydrogel showed stronger bactericidal properties than the other two MOFs (Figure 8), mainly due to a stable 3D framework with a higher surface area to volume ratio than the other MOFs investigated. They realized the much more significant impact of the central metal and the structure of the MOF over the type of ligand in determining antibacterial effects (Figure 9).

Their hydrogels exhibited swelling ratios 20 times greater than that of the control hydrogel. Yet, it also lowers the MOF concentration more than 20-fold after encapsulation compared to the free MOF, resulting in a significant decrease in the antibacterial activity of the hydrogels (Figure 8 and Figure 9). Furthermore, direct contact between the MOF and the bacterial membrane was blocked by steric hindrance of the MOF-containing hydrogels, disturbing the antibacterial efficiency of the MOFs and resulting in highly reduced bactericidal effects of MOF-embedded hydrogels compared to free MOFs. However, they still observed better antimicrobial activities than some of the previously reported antibacterial hydrogels in the literature thanks to the high surface area to volume ratio derived from the 3D framework of Cu-based MOF-embedded hydrogel and the natural antibacterial property of copper.

**Figure 10:**
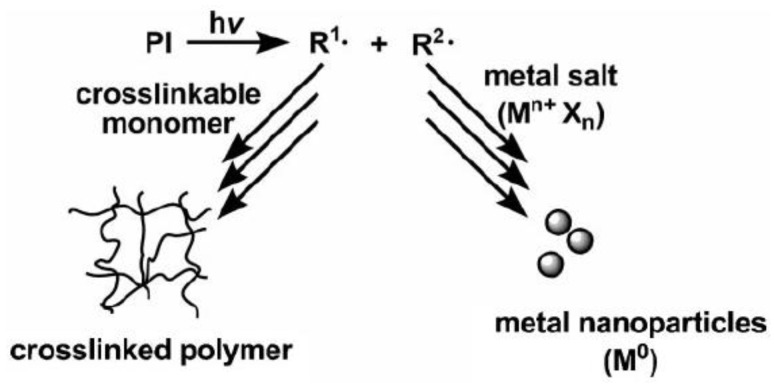
In situ synthesis of polymer network containing metal nanoparticles (NPs) by light-induced processes. Reprinted with permission. Copyright 2009, Wiley [65].

Other groups of scientists also focused on the synthesis of antibacterial hydrogels containing bactericidal agents, such as silver NPs, via free radical photopolymerization. Uygun et al. reported the synthesis of such material using photoinduced free radical polymerization [65]. In their study, they described an in situ synthesis of an acrylamide-based hydrogel loaded with silver NPs using on-site precipitation of silver salt to metal NPs and simultaneous polymerization of acrylamide monomers and crosslinkers afforded by the photolysis of a benzoin-type photoinitiator (PI). The latter generates free radicals upon irradiation, simultaneously inducing the polymerization and the salt reduction to metal NPs (Figure 10).

For the synthesis of their hydrogels, they mixed nitrogen-flushed samples of acrylamide, bisacrylamide, AgNO_3_ and citrate with photoinitiator Irgacure 2959 in various quantities before irradiating them at 350 nm for 15 min at room temperature. They further investigated water uptake, thermal stability and the porosity of the structures of the gels alongside their antibacterial activities against both pathogenic and nonpathogenic *E. coli* and *S. aureus*.

They reported a homogeneous size distribution of the silvers NPs that were incorporated and stabilized within a highly crosslinked hydrogel network thanks to citrate and noticed that their incorporation did not affect the honeycomb-like morphology and pore size of the hydrogel. Moreover, they observed greater water uptake than that of pure hydrogel due to citrate, which interacts more with water and enhances the hydrophilicity of the hydrogels, thus showing promising water-based applications. Silver NP-containing hydrogels featured better thermal stability and high antibacterial activity against both types of *E. coli* bacteria. However, they exhibited a lower bactericidal effect against the *S. aureus* strain due to a facilitated interaction between NPs and the cell wall of Gram-negative bacteria. Owing to the relative abundance of negative charges on the cell membranes of such types of bacteria, silver NPs can easily penetrate the cell and strongly interact with the cellular components, causing efficient bacterial growth inhibition.

**Figure 11:**
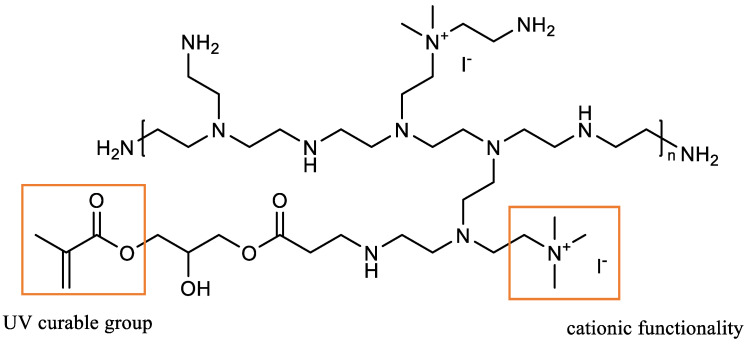
Chemical structure of modified UV-curable cationic poly(ethyleneimine) (Quv-PEI).

**Figure 12:**
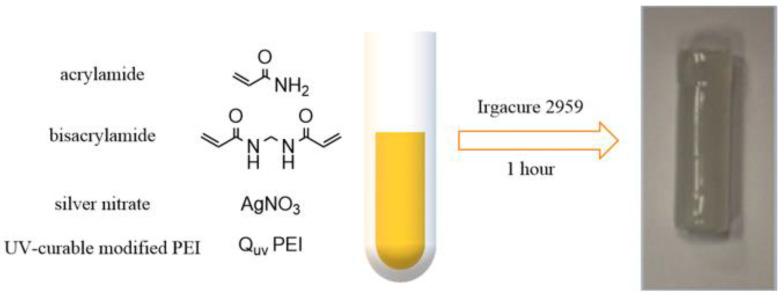
Schematic representation of the photoinduced formation of UV-curable cationic poly(ethyleneimine) (Quv-PEI)-based silver-loaded hydrogel. Reprinted with permission. Copyright 2016, Elsevier [44].

In another study, Palantoken et al. reported the use of modified cationic PEI for the development of a novel antimicrobial hydrogel loaded with silver NPs as well, acting thus on a dual bactericidal mechanism and with potential long-lasting antibacterial activity toward both Gram-positive and Gram-negative bacteria [44]. They described the addition of methacrylate functions on the free amine substituents of the PEI polymeric backbone using 3-(acryloyloxy)-2-hydroxypropyl methacrylate in ethanol under a nitrogen atmosphere before obtaining a UV-curable cationic PEI (Q_uv_-PEI) by mixing methacrylated PEI and methyl iodate in the same conditions (Figure 11). They obtained the Q_uv_-PEI-based silver-loaded hydrogels by stirring acrylamide, bisacrylamide and silver nitrate AgNO_3_ in different amounts with Q_uv_-PEI in water (Figure 12). On-site precipitation was used to incorporate the silver-based biocidal agent within the polymeric network, the reduction of silver salt to silver being afforded by the UV-curable system (Figure 10). After adding photoinitiator Irgacure 2959 to the mixture, it was irradiated for one hour at room temperature at 300 nm to afford a free radical photo-crosslinking copolymerization. They assessed the cytotoxicity in vitro as well as determined the dual antibacterial effects of the studied hydrogels against *E. coli* and *S. aureus* using airborne testing and Kirby–Bauer disk diffusion method.

Their results indicated high antibacterial effects on surfaces and in a solution of the resulting hydrogels toward both bacterial strains tested that were due to the dual action of membrane disruption afforded by the presence of cationic PEI at the surface of the hydrogels and the biocidal effects of leached Ag^+^ ions. This antibacterial effect was, however, higher against *E. coli* than *S. aureus*, which they also explained by the difference in thickness and composition of the external structures of the bacterial membranes between the Gram-positive and Gram-negative bacteria. They demonstrated in vitro biocompatibility of their hydrogels, despite a sustained release of silver ions in aqueous media. The leaching rate of Ag/Ag^+^ ion could otherwise be controlled by varying the concentration of AgNO_3_ in the hydrogel formulation.

These last studies demonstrated that NPs of silver play thus a dual antibacterial role: for colloidal silver NPs, the bactericidal action would be due to the formation of Ag^+^ cations from the NPs by an oxidation reaction, given that Ag^+^ ions disrupt bacterial cell membranes and inhibit the enzymatic activity of bacterial cells [43]. In addition, silver NPs themselves feature antibacterial properties; they could, however, be harmful to host tissues, making it of the utmost importance to prevent the release of silver NPs within infected areas.

If the last two studies introduced silver NPs via an in situ approach, by reducing Ag^+^ cations into metal NPs during the crosslinking step of the polymerization, ensuring a homogeneous dispersion of NPs within hydrogels, Zakia et al. used an ex situ approach, consisting of the mixing of presynthesized silver NPs and monomer units before the crosslinking [43]. To prevent NP agglomeration from occurring during the crosslinking process, reducing hence the antibacterial efficacy of silver NPs, their surface can be modified, by proteins such as collagen, for instance, to enhance stabilization within hydrogel networks before gelation occurs [83].

**Figure 13:**
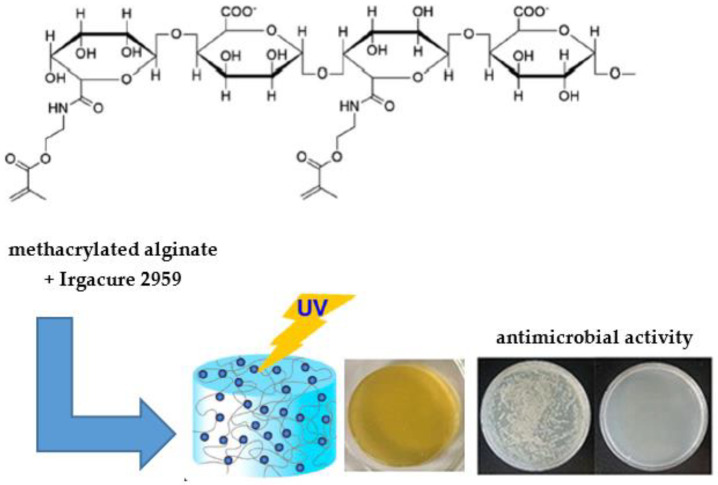
Illustration of the preparation of a methacrylated-alginate-based hydrogel using free-radical photopolymerization in a silver nanoparticle aqueous solution and its antibacterial activity (left: hydrogel without silver NPs; right: hydrogel obtained using a 1.5 nM silver NP aqueous solution). Reprinted with permission. Copyright 2020, Taylor and Francis Online [43].

In their study, Zakia et al. investigated alginate-based hydrogels loaded with ex situ synthesized silver NPs. To prevent NP agglomeration in the presence of silver NPs upon the addition of divalent cations, such as Ca^2+^, for the classic cation-assisted crosslinking of the linear brown algae derived-polysaccharide, they aimed for a photo-crosslinking process afforded by the introduction of methacrylate functions onto the alginate backbone using a carbodiimide agent. They obtained an alginate-based hydrogel featuring a homogenous NP distribution within its network by dissolving the methacrylated alginate and photoinitiator Irgacure D2959 in the silver NP aqueous solution, which they irradiated with a 365 nm UV light for 15 min (Figure 13). They tested the resulting hydrogels against *E. coli* for antibacterial assays.

They reported the size and shape homogeneity of the synthesized silver NPs with a noticeable stable colloidal state, probably due to citrate molecules, alongside a uniform dispersion of Ag NPs within the polymer network of the gels. They also observed a silver NP dose-dependent antibacterial activity of their hydrogels, as their results range from a negligible bactericidal effect for an NP concentration of 0.5 nM to complete bacterial growth inhibition for an NP concentration of 1.5 nM. This behavior can be explained by the release of Ag^+^ cations from the hydrogel, whose effect is restricted when the NP concentration is low because of the favorable interaction between Ag^+^ ions and the -COO^−^ groups on the polymeric alginate chain. Working on the preparation of micro-/nanosized hydrogels would eventually provide a larger surface area, facilitating thus the release of silver cations into the medium, but it would also decrease the antibacterial activity of the system due to a fast release rate. The goal achieved is to exert long-term effective bactericidal effects via the control of the release of Ag^+^ cations by adjusting the hydrophilicity of the hydrogel network through its chemical nature and physical structure.

**Figure 14:**
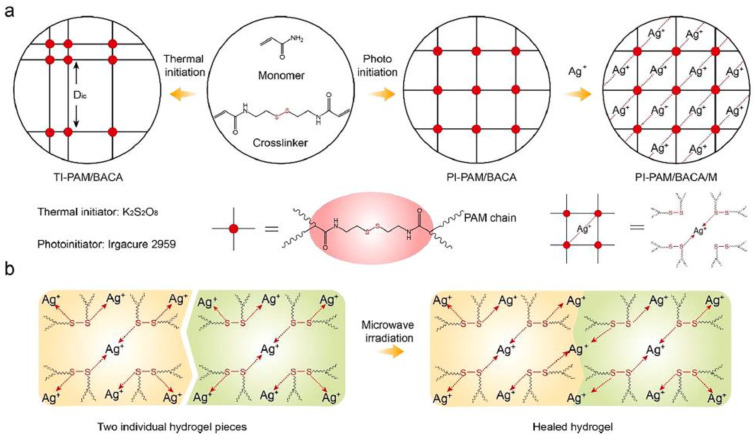
(**a**) Illustrations of the preparation of AM/BACA-based hydrogels using either thermal or photoinduced free-radical polymerization. The introduction of silver cations in the photoinduced network was achieved through a deswelling/swelling process. (**b**) Illustration of the healing mechanism of photoinduced hydrogel under microwave irradiation. Reprinted with permission. Copyright 2021, Elsevier [84].

In another study conducted by Chen et al. on metal-ion-loaded photoinduced hydrogels as bio-interactive dressings, the group of scientists demonstrated that the hydrogels they investigated featured far greater mechanical properties when obtained via a photochemical process rather than by conventional thermal methods [84]. In their work, they reported how they obtained a hydrogel with a well-defined network morphology and an optimal healing efficiency of 85% in 30 s, alongside highly stretchable and mechanically robust properties, using an organic crosslinking agent containing a UV-responsive disulfide bond. When exposed to external stimuli such as UV light, disulfide derivatives are prone to cleavage of the S-S and the C-S bonds, producing thiyl or perthiyl radicals. Chain exchange reactions allow then the formation of energetically favorable structures [85]. By integrating disulfide bonds into the free-radical polymerization crosslinking agent, Chen et al. expect to optimize the potential irregular hydrogel network in order to get a well-distributed polymeric structure. To do so, they dissolved acrylamide (AM), N,N′-bis(acryloyl), cysteamine (BACA) and Irgacure 2959 in water in ultrasonication conditions for 5 min. After flushing the atmosphere with nitrogen, they irradiated their mixture for 20 min at 365 nm before adding the resulting photoinduced hydrogel (PI-PAM/BACA) in their silver salt aqueous solution to achieve their metal ion loaded gel (PI-PAM/BACA/Ag) using a deswelling/swelling method (Figure 14a). In the scope of their study, they also prepared a control sample of their hydrogel through thermal polymerization (TI-PAM/BACA). The antibacterial action of the investigated gels was tested against *E. coli*, *S. aureus* as well as *Candida albicans* (CA), and in vitro biocompatibility assays as well as in vivo wound healing applications were also studied.

Direct comparisons between photoinduced and thermally initiated networks were studied throughout the different analyses. Thorough imaging analysis indicated that the photo-initiated networks showed a well-distributed honeycomb-like polymeric structure with uniform pore sizes and homogenous Ag^+^ distribution, whereas the thermally induced networks displayed a broad distribution in pore morphology, ranging from 200 nm to 5 µm. The evenly distributed polymeric network afforded by photopolymerization exhibited excellent stretchable and elastic behavior, undergoing high-level deformations such as twisting, knotting, extensive stretching (>2500%), high compressions, and notches and slices with a blade without breakage. In addition, the introduction of Ag^+^ cations in the polymer structure containing disulfide bridges generated the formation of Ag^+^-disulfide metal coordination interactions, giving the material highly efficient healing properties (Figure 14b), and the healed samples still presented highly efficient stretchable properties as compared with those of their original samples. The introduction of metal ions also enabled the improvement of mechanical strength, notch insensitivity and elastic behavior via the efficient energy dissipation from dynamic metal crosslinks. On the contrary, uneven networks obtained using conventional thermal methods gave brittle structures.

Antibacterial activity and biological compatibility assays carried out on the photoinduced hydrogels demonstrated a positive relationship with the concentration of silver cations introduced as well as good biocompatibility. In vivo studies showed a great affinity of the investigated hydrogel on wound surfaces alongside improved wound healing performances, thanks to efficient bactericidal effects from Ag^+^ ions and moisture features of the hydrogels.

### 3.2. Light-Triggered Antibacterial Activity of Hydrogels

**Figure 15:**
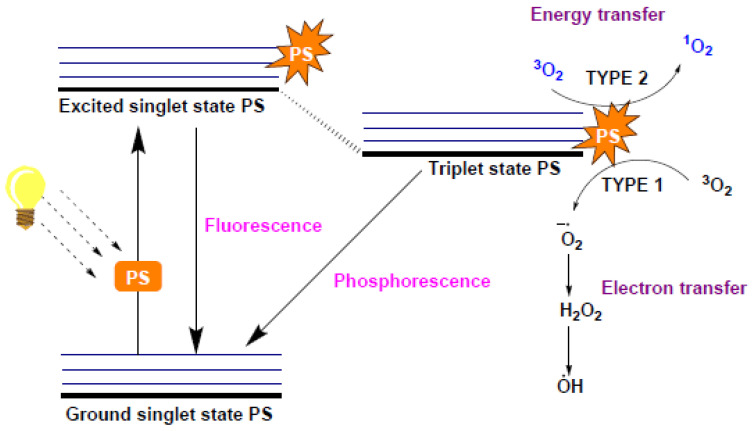
Schematic illustration of the generation of reactive oxygen species (ROS) via the transfer of excitation energy between a photosensitizer in its excited triplet state and molecular oxygen under irradiation. Reproduced by permission of The Royal Society of Chemistry (RSC) on behalf of the European Society for Photobiology, the European Photochemistry Association, and RSC [34].

When it comes to hydrogels for which antibacterial properties are light-sensitive, their mechanism of action falls in line with photodynamic therapy (PDT). On this topic, several most interesting reviews dealing with PDT, hydrogels and their use in medicine have been published [34,53]. Briefly, the antibacterial mechanism of action is based on the generation of reactive oxygen species (ROS) because of an energy transfer from a light-absorbing chromophore-based compound called photosensitizer (PS) in its excited triplet state to molecular oxygen (Figure 15) [34,53,63,86]. The photosensitizer goes from its fundamental electronic singlet state to a short-lived excited singlet state (^1^PS*) upon irradiation under an adequate wavelength. The photosensitizer either reverts back to its ground state, thus emitting the corresponding energy via fluorescence, or sustains intersystem crossing to an excited triplet state (^3^PS*), which is sufficiently long-lived to undergo different chemical reactions or to enable the transfer of excitation energy to another compound such as triplet oxygen [^3^O_2_]. It consequently generates cytotoxic reactive oxygen species or gives rise to singlet oxygen [^1^O_2_], a highly reactive form of oxygen, all of which induces damage to cells or bacteria [87]. From the excited triplet state, the photosensitizer goes back to its ground electronic state via phosphorescence.

Depending on the reactive species formed, two types of PDT can be distinguished [34,53,63,86]. Redox reactions between ^3^PS* and substrates in the studied environment leading to the generation of radicals, which ultimately interact with molecular oxygen and other compounds in the environment to form oxygen, peroxide or hydroxide radicals, constitutes type I. Type II relies on the direct interaction between ^3^PS* and molecular oxygen, generating singlet oxygen [^1^O_2_], which is able to diffuse within cellular matrices and to induce apoptosis by denaturing DNA/RNA or proteins via the oxidation of their constituents.

For hydrogels for which antibacterial properties are based on the light-triggered generation of ROS or [^1^O_2_], photosensitizers are usually included in the preparation mixture and embedded in the final 3D polymeric network, either by grafting them onto oligomeric backbone units [42], by covalently conjugating them with monomeric units during crosslinking [87] or by encapsulating them within the hydrogel crosslinked network [88]. Upon irradiation, such a hydrogel is thus able to generate ROS or [^1^O_2_].

The following studies focused their energy on the investigation of photosensitive antibacterial hydrogels, integrating thus the principle of PDT at the heart of their work via the incorporation of photosensitizers and other substances with excellent photocatalytic activities into their hydrogels for antibacterial PDT applications.

#### 3.2.1. Light-Sensitive Antibacterial Hydrogels Loaded with Metal Nanoparticles

Mao et al. recently reported the synthesis of a hydrogel embedded with Ag/Ag@AgCl/ZnO nanostructures featuring a photo-inspired antibacterial activity [88]. Their composite hydrogel is based on carboxymethyl cellulose (CMC), a naturally occurring biocompatible polysaccharide with good solubility and high chemical stability, and Ag/Ag@AgCl/ZnO hybrid nanostructures embedded in the polymeric network. Zinc oxide is a semiconductor nanomaterial exhibiting an excellent photocatalytic activity as well as antibacterial properties via the generation of ROS, the release of zinc ions from ZnO or by direct contact with ZnO particles [89]. Upon exposure to light, the photocatalytic reaction of ZnO NPs leads to the generation of hydroxyl radicals, superoxide radicals and singlet oxygen, which cause serious damages to cellular constituents such as lipids, proteins and DNA. Additionally, the incorporation of metal NPs such as silver on ZnO nanostructures not only significantly enhances the antibacterial efficacy of ZnO particles but also adds bactericidal properties of these metal NPs [88,89]. It also prevents the recombination of photoexcited electrons and protons generated along with the ROS, which tends to reduce the photocatalytic activity of ZnO, hence reducing its antimicrobial activity along with it.

**Figure 16:**
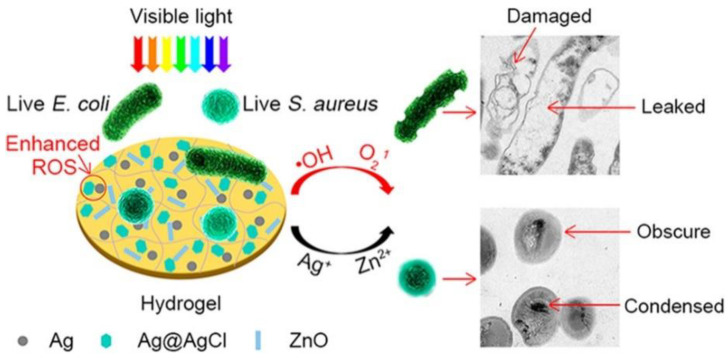
Schematic illustration of the antibacterial action of the Ag/Ag@AgCl/ZnO nanocomposite hydrogel under visible light irradiation on *E. coli* and S. *aureus* bacteria strains. Reprinted with permission from ACS Nano 2017, 11, 9, 9010–9021. Copyright 2017, American Chemical Society [88].

In their study, Mao et al. prepared their hydrogels by adding epichlorohydrin to CMC previously dissolved in a basic aqueous solution under continuous stirring. They afforded their prepolymer by heating the stirred mixture to 80 °C for 2 h. The metal embedded hydrogels were obtained by immersing the swollen gel samples in solutions of silver nitrate (AgNO_3_) before illuminating them with UV light (365 nm) for 2 h to afford the reduction of silver cations to Ag NPs. ZnO nanostructures were then incorporated by successively immersing the Ag/Ag@AgCl embedded gels previously obtained in a solution of zinc nitrate (Zn(NO_3_)_2_) and in a solution of sodium hydroxide followed by freeze-drying overnight to obtain the Ag/Ag@AgCl/ZnO nanocomposite hydrogels (Figure 16).

Mao et al. tested their hydrogels against *E. coli* and *S. aureus*. Although they observed clear inhibition zones for their nanocomposite hydrogels used as such, attesting to good antibacterial effects, they noticed a significant increase of bactericidal effect after irradiating their samples with simulated sunlight for 20 min, certainly due to the formation of ROS resulting from the photoexcitation of ZnO. Their results showed broad antibacterial activity against both bacteria strains tested as well as accelerated wound healing properties. In addition, the light-sensitive antibacterial activity of metal embedded hydrogels is enhanced by metal cations leaching from silver and zinc NPs. Through electrostatic forces, metallic cations can strongly adsorb to the cell surface of bacteria and cause the destruction of charge balance and the deformation of the cells up until the death of the bacteria. They can also interact with functional groups of cellular proteins after entering the cell and lead to the death of the bacteria due to unbalanced metabolism [89]. Direct contact with ZnO particles may also play a role in the overall antibacterial activity of the hydrogels considered in this study.

Recently, hydrogels have therefore been studied as carriers for photosensitizers. Furthermore, working on zinc-containing structures, Bayat et al. described the development of a chitosan-based hydrogel using a photosensitizer as a crosslinker, zinc phthalocyanine tetra-aldehyde (ZnPcTa), conjugated with colistin [87]. When exposed to visible light, the excited photosensitizer covalently attached to the hydrogel network is able to transfer its energy to molecular oxygen, resulting thus in the formation of radicals and singlet oxygen, deadly species for bacteria.

**Figure 17:**
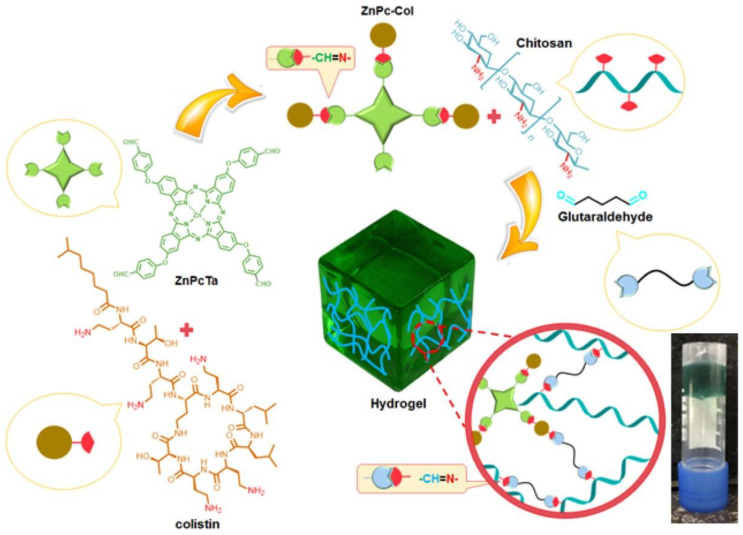
Schematic representation of the ZnPc–Col-containing hydrogel. Reprinted with permission. Copyright 2019, Elsevier [87].

In order to enhance zinc phthalocyanine (ZnPc) bioavailability and solubility in aqueous solution, as well as the efficiency of the photo-inspired antibacterial activity (also called antibacterial photodynamic therapy (aPDT)), Bayat et al. investigated the incorporation of a ZnPc–colistin conjugate within their chitosan-based hydrogel network (Figure 17). Colistin is an amphiphilic antibacterial polypeptide exerting its bactericidal action via the disruption of the bacterial outer membrane and is particularly active against Gram-negative bacteria [90]. In the scope of their study, Bayat et al. prepared their ZnPc–colistin conjugate by successively adding an aqueous solution of NaOH and a solution of ZnPcTa in DMF in an aqueous solution of colistin sulfate, in a molar ratio of 1:3 of ZnPcTa and colistin. They left their mixture under continuous stirring for four days to afford the desired conjugate. To obtain the covalently crosslinked hydrogel, they added solutions of ZnPc–colistin conjugate and glutaraldehyde in acetic acid to a solution of chitosan also dissolved in acetic acid, and gelation occurred upon shaking. They investigated the morphology, mechanical properties as well as photodynamic antibacterial properties of the resulted in hydrogels against *Pseudomonas aeruginosa* and studied the generation of singlet oxygen to assess their photocatalytic activity.

They observed a sponge-like interconnected porous network with tunable pore size and viscoelastic properties controlled by the amounts of ZnPc–colistin conjugate and glutaraldehyde used for the synthesis.

The bactericidal effect, hence, the generation of singlet oxygen, occurred upon release of the ZnPc–colistin conjugate from the hydrogel matrices, which again can be tuned by controlling the crosslinking degree with glutaraldehyde—a higher crosslinking degree induced a slower release rate of phthalocyanine, meaning lower significant antibacterial activity and singlet oxygen production. Hydrogels based on modified ZnPc–colistin conjugates exhibited improved bactericidal effect under light against the bacteria strain tested, whereas chitosan-based hydrogels containing ZnPcTa showed no antibiotic activity during tests under both dark and light conditions. The binding of the photosensitizer to the bacterial membrane is required for the photodynamic antibacterial effect to occur, and it appears that ZnPcTa alone is not able to permeate on outer bacterial membranes due to a lack of cationic unit. Overall, they obtained a biocompatible chitosan-based hydrogel incorporated with zinc phthalocyanine–colistin conjugates exhibiting enhanced antibacterial activity under light due to the photocatalytic generation of singlet oxygen and showing promises for potential use as wound dressings.

#### 3.2.2. Organic Light-Sensitive Antibacterial Hydrogels

Another group worked on photodynamic inactivation of bacteria causing inflammatory skin lesions, using chitosan-based hydrogels incorporated with a photosensitizer for aPDT applications [91]. Chitosan is once again an alternative of choice in this study, as it is a biocompatible and biodegradable biomaterial exhibiting intrinsic antibacterial and anti-inflammatory properties as well as the ability to serve as a vehicle for active compounds. In their study, Frade et al. worked on the in vitro aPDT efficacy against *Propionibacterium acnes* in planktonic and biofilm phases of a chitosan-based hydrogel containing methylene blue (MB) as a photosensitizer to be irradiated under a red light (660 nm). *P. acnes* bacterium is one of several factors whose interaction results in the development of acne, a common disease causing non-inflammatory to inflammatory skin lesions. In addition, it is known to form a biofilm, a cellular organization in which cells are more resistant to antibacterial agents compared to bacterial suspensions. In their work, Frade et al. prepared their chitosan hydrogel by dispersing chitosan in acetic acid solutions under magnetic stirring for 24 h before adding poloxamer 407 under continuous agitation. To obtain their MB-loaded hydrogel, they finally incorporated MB at different concentrations to the previously obtained gels. They studied rheological properties of their chitosan-based samples and compared in vitro bactericidal effects against *P. acnes* in both planktonic and biofilm phase of a chitosan hydrogel alone, of an aqueous solution of MB associated with a red LED system alone, and of a chitosan-based hydrogel incorporated with MB under the same irradiating system for assessment of aPDT.

**Figure 18:**
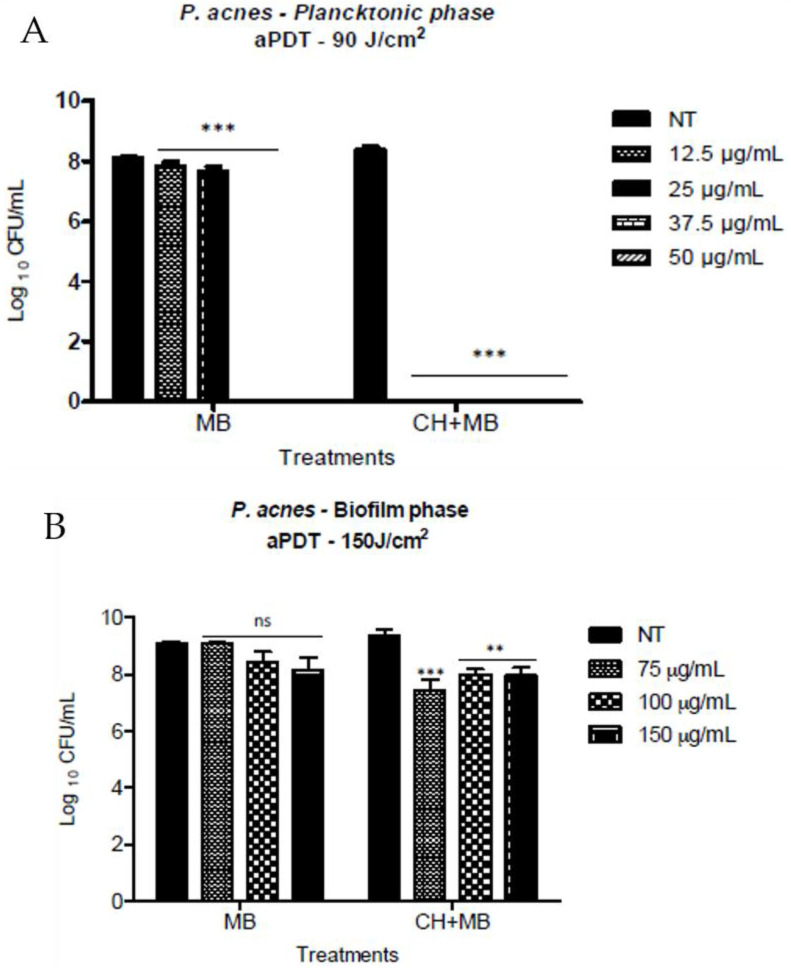
Antibacterial photodynamic therapy (aPDT) mediated by methylene blue (MB) in solution and by MB incorporated into 0.25 chitosan hydrogel over the standard suspension of *P. acnes* (**A**) or biofilm of *P. acnes* (**B**). Columns represent the average of three independent assays, and bars represent the standard deviation. The asterisks indicate where there is a statistical difference in comparison with the groups treated and the group non-treated (NT); (two-way ANOVA with posttest Bonferroni), ** *p* < 0.01; *** *p* < 0.001. Reprinted with permission. Copyright 2018, MDPI [91].

They observed bacterial reduction for chitosan evaluated separately on both bacterial phases tested, although without complete elimination of the bacterial strain, highlighting thus natural antibacterial properties of this polysaccharide. Their results also showed bacterial reduction ranging from slight bactericidal effects for low concentrations of MB in solution to complete bacterial elimination for concentrations of MB of 37.5 µg. mL^−1^ and higher against *P. acnes* in bacterial suspensions (Figure 18A). However, no significant bacterial reduction was obtained using aqueous solutions of MB for mediated aPDT against *P. acnes* biofilm for all the MB solutions evaluated (up to 150 µg. mL^−1^; Figure 18B). Concerning tests conducted on a chitosan-based hydrogel incorporated with MB, not only total microbial reduction was achieved against *P. acnes* bacterial suspensions, but also there seems to be a synergistic effect of aPDT with chitosan as total bacterial reductions were achieved with concentrations in MB three-fold lower when compared to aPDT with MB alone. As for aPDT effects of chitosan hydrogel loaded with MB against *P. acnes* biofilm, all three concentrations tested exhibited enhanced antibacterial properties when compared to MB solutions alone but were nevertheless similar to that observed when the biofilm was treated with chitosan hydrogel alone, hence ruling out the synergistic effect possibility.

#### 3.2.3. Hybrid Organic–Inorganic Light-Sensitive Antibacterial Hydrogels

In the framework of using dyes as photosensitizers, Li et al. studied the introduction of rose bengal (RB), a well-known dye and photosensitizer, into graphene oxide (GO) based-hydrogel matrix in order to benefit from a synergistic antibacterial effect of photothermal therapy and photodynamic therapy [42]. They described the development of a hybrid hydrogel based on GO modified with a β-cyclodextrin aldehyde (β-CD-DA), RB grafted on chitosan microspheres via covalent immobilization and poly(vinyl alcohol) (PVA), with the purpose of obtaining a biocompatible and antibacterial material for wound dressing applications

**Figure 19:**
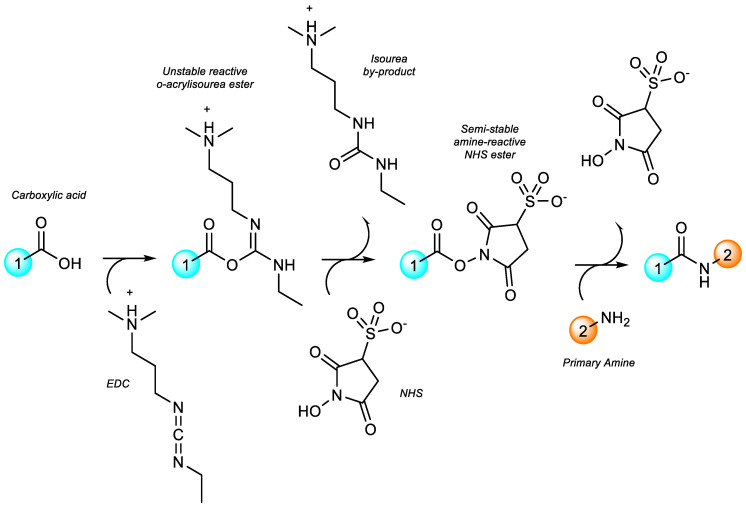
General reaction mechanism of the conjugation of a carboxylic acid-containing substrate with a primary amine functional group via covalent immobilization using *N*-ethyl-*N*′-(3-dimethyl aminopropyl)carbodiimide (EDC) and *N*-hydroxysuccinimide (NHS).

For the preparation of their hydrogel, they started by modifying GO with β-CD-DA to get β-GO, a multipurpose inorganic network intertwined with the porous hydrogel structure, simultaneously fixing GO nanosheets and preventing the release of GO NPs. They dispersed GO in 80% ethanol using an ultrasonic bath and added (3-aminopropyl) triethoxysilane to the GO dispersion. After a washing step using anhydrous ethanol and a centrifugation step, they obtained a GO-NH_2_ precipitate, which was dispersed in DMSO using ultrasonic methods. A uniform β-GO solution was obtained by adding β-CD-DA to the GO-NH_2_ dispersion solution under continuous stirring. In parallel, they prepared chitosan microspheres (CM) by adding tripolyphosphate to a stirred chitosan solution. After a centrifugation step, the supernatant was removed, and the resulting solids were freeze-dried. To obtain chitosan microspheres grafted with RB, they added previously obtained CM to RB chemically modified with *N*-ethyl-*N*′-(3-dimethyl aminopropyl) carbodiimide (EDC) and *N*-hydroxysuccinimide (NHS) to perform an EDC/NHS covalent immobilization (Figure 19). Finally, they added CM grafted with RB and PVA to the β-GO dispersion solution, which was then degassed under vacuum before undergoing 4 freeze–thaw cycles to obtain the final hybrid β-GO/RB/PVA hydrogel. They also prepared PVA, GO/PVA and β-GO/PVA hydrogels to serve as a reference. Within the scope of wound dressing applications, they analyzed the mechanical properties, swelling behavior, biocompatibility as well as wound healing capabilities of the resulting gel. They assessed in vitro photothermal effects, ROS generation, in vitro and in vivo photodynamic antibacterial properties against *E. coli* and *S. aureus* of the hydrogel samples.

**Figure 20:**
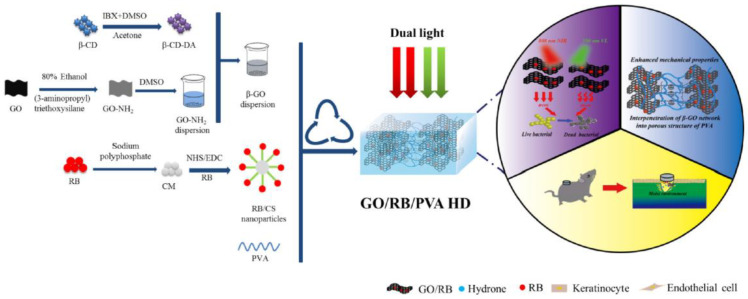
Schematic illustration of the synthesis of the β-graphene oxide (GO)/ rose bengal (RB)/poly(vinyl alcohol) (PVA) hydrogel and the promotion mechanisms of mechanical property, antibacterial activity and wound healing. Reprinted with permission. Copyright 2021, Elsevier [42].

Thanks to the addition of a modified graphene oxide inorganic network within a PVA porous structure, the mechanical properties of the resulting hydrogels are enhanced, although the addition of RB grafted on CM seems to reduce compressive properties. Li et al. also reported a good water-absorbing capacity of the composite hydrogel. Concerning photothermal effects, the hybrid hydrogel exhibits an excellent photothermal conversion capacity under an 808 nm light irradiation with no significant changes when compared to non-modified GO/PVA reference hydrogel, suggesting that photothermal properties of GO remain unchanged, even in the presence of RB and if GO has been chemically modified. In terms of the generation of ROS, they observed the production of hydroxide radicals under irradiation at both 550 and 808 nm, with the best result under dual light illumination. Singlet oxygen [^1^O_2_] is generated by the hybrid hydrogels only under irradiation at 550 nm and under the dual light illumination. Finally, they noticed that the chemical modification of GO decreases the photocatalytic activity of the hydrogels, which is, however, greatly improved by the introduction of RB. Regarding antibacterial effects, the hybrid hydrogel benefits from a synergistic bactericidal effect of hyperthermia brought by GO under irradiation at 808 nm, combined with a small generation of hydroxide radicals and ROS generated by RB under irradiation at 550 nm. The best antibacterial results are obtained under a dual light illumination over a 10 m time period, combining thus both bactericidal mechanisms, with an antibacterial efficiency against *E. coli* and *S. aureus* of 99.3% and 97.7%, respectively. In vivo experiment outcomes showed consistency with in vitro results, with the composite hydrogel presenting a fast healing capacity and rapid quenching of the inflammatory response. Overall, Li et al. obtained a biocompatible and biosafe modified graphene oxide-based hydrogel incorporated with rose bengal grafted on chitosan microspheres, featuring excellent antibacterial properties (Figure 20).

**Figure 21:**
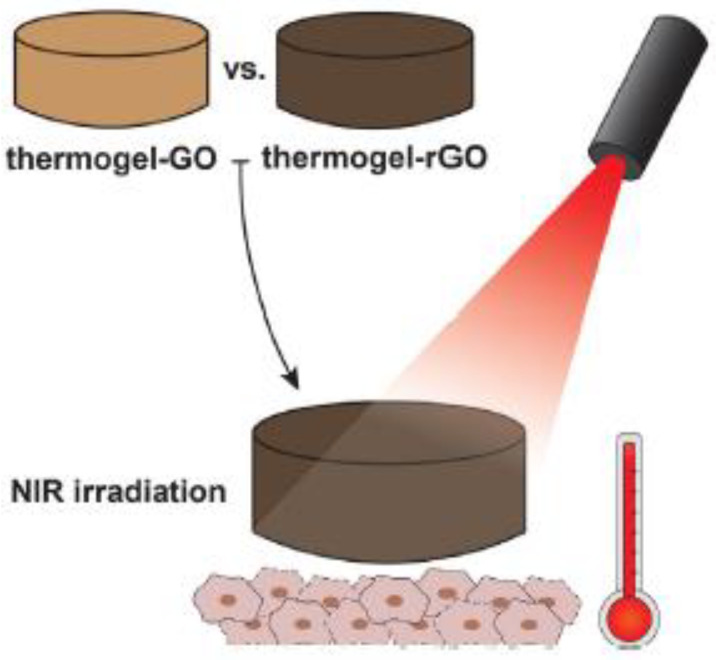
Schematic representation of photothermal therapy (PTT) mediated by thermogel–GO and thermogel–rGO. Reprinted with permission. Copyright 2020, Elsevier [92].

Also working on GO-based hydrogels, Lima-Sousa et al. investigated graphene oxide and reduced graphene oxide (rGO) in the scope of cancer photothermal therapy (PTT) and near-infrared (NIR) light-enhanced antibacterial applications [92]. The PTT rests on a similar principle to PDT: a NIR light irradiation on particular materials capable of absorbing such beams produces a harmful temperature increase for cancer cells (Figure 21). In their work, they studied a formulation of chitosan and agarose containing GO or rGO for injectable thermo-responsive in situ hydrogel formation in the framework of GO/rGO local deliveries at tumor sites while avoiding rapid clearance from blood circulation. They chose to work with natural and biocompatible polymers such as chitosan for its natural antibacterial effect, while agarose was chosen to mediate a thermo-responsive crosslinking. They prepared their hydrogel samples by mixing heated solutions of agarose dissolved in water with chitosan dissolved in 1% acetic acid solutions. In parallel, rGO was obtained by treating GO with L-ascorbic acid. They mixed GO and rGO nanoparticles with agarose-chitosan solutions before an extrusion step, enabling them to afford hydrogels with uniform macroscopic features. They finally characterized the physicochemical properties, biocompatibility, photothermal effects as well as the light-mediated antibacterial activities against *E. coli* and *S. aureus* of their hydrogel samples.

**Figure 22:**
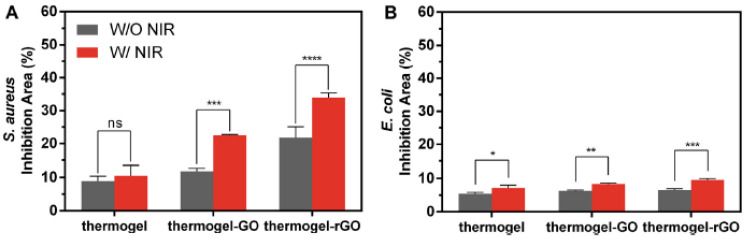
Characterization of the near-infrared (NIR)-light enhanced antibacterial activity of the different hydrogel formulations. Analysis of the inhibition area obtained for thermogel, thermogel–GO and thermogel–rGO in contact with *S. aureus* (**A**) or *E. coli* (**B**) without NIR (W/O NIR) or with NIR (W/ NIR) laser irradiation (808 nm, 1.7 W.cm^−2^, 10 min). Values are presented as mean ± SD, n = 3. (* *p* < 0.05, ** *p* < 0.01, *** *p* < 0.001, **** *p* < 0.0001, ns = non-significant). Reprinted with permission. Copyright 2020, Elsevier [92].

The resulting GO and rGO-based hydrogels featured highly porous interconnected inner structures with uniform and well-ordered networks. The introduction of graphene nanomaterials to the hydrogel formulations did not affect their gelation time, their injectability capacity, nor did it affect their cytocompatibility. The photothermal capacity of the samples was investigated by exposing the thermo-hydrogels to NIR-light for 10 min (808 nm) while measuring the temperature. GO-based hydrogels generated a 2.2 °C temperature increase, whereas rGO-based hydrogels produced a temperature increase of 8.1 °C, underlining promising potentials for reduced graphene oxide in cancer PTT. Their tests showed a breast cancer cell viability reduction to about 60% after irradiation with a NIR light at 808 nm for 10 min. As for antibacterial effects, the thermo-gels exhibited natural bactericidal activities thanks to chitosan being incorporated within their matrices, with stronger effects against *S. aureus* than against *E. coli* (Figure 22). In addition, the irradiation of the graphene-based thermo-gels with NIR light (808 nm for 10 min) further enhanced bactericidal effects, which Lima-Sousa et al. explained by the so-generated heat causing a possible chitosan dissolution from the polymeric matrix. Ultimately, they reported a stable, biocompatible and biodegradable thermo-responsive graphene-based hydrogel with suitable physicochemical properties to be studied as in situ forming injectable polymeric matrices for light-mediated biomedical applications.

In the area of aPDT, the most classically studied photosensitizers are porphyrins and their derivatives, a class of hydrophobic and heterocyclic organic photosensitizers composed of four pyrrole subunits [34]. Porphyrins are naturally occurring compounds since they are key constituents for the respiratory system in living organisms [53,86]. In their investigation, Kumari et al. described the synthesis of a hybrid hydrogel based on DNA containing carbon dot (CD) nanoparticles and protoporphyrin IX (PpIX), a photosensitizer for potential sustained aPDT applications [93]. Porphyrins constitute the elementary chemical structure of heme, the blood pigment responsible for iron homeostasis in living cells and co-factor in the biosynthesis of hemoglobin [53,86]. The most widespread heme-type is b-heme, also known as iron–protoporphyrin IX [94]. Carbon dot refers to carbon nanoparticles smaller than 10 nm, which were fortuitously discovered in 2004 throughout the purification of single-walled carbon nanotubes [95,96]. They consist of fluorescent carbon nanomaterials with amorphous to nanocrystalline cores and abundantly functionalized surfaces.

**Figure 23:**
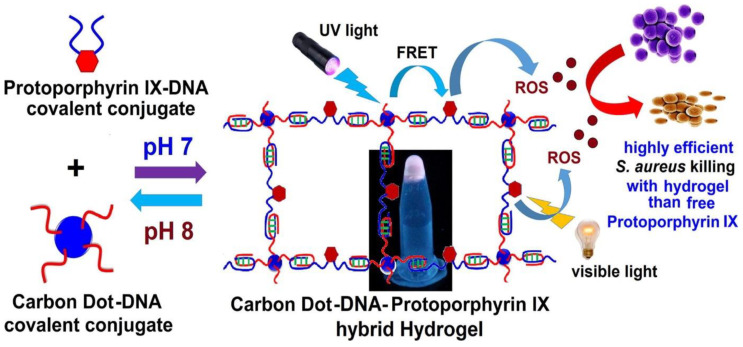
DNA-carbon dot–protoporphyrin IX hybrid hydrogel formation and its antibacterial photodynamic therapy applications. Reprinted with permission. Copyright 2019, Elsevier [93].

In their work, Kumari et al. investigated CD as both a crosslinker and as a way to incorporate fluorescent-based tracking while improving the PpIX-mediated ROS generation capacity of the DNA-based hydrogel through Forster resonance energy transfer (FRET) for advanced aPDT applications [93]. Succinctly, they covalently immobilized ethylene diamine on PpIX using an EDC/NHS synthetic pathway (Figure 19). They then conjugated PpIX–ethylene diamine with phosphorylated cytosine-rich single-stranded DNA (ssDNA) via an EDC/1-methyl imidazole activation step. CD containing surface primary amine functional groups were concomitantly conjugated with cytosine-rich ssDNA through the same EDC/imidazole chemistry. Finally, the hybrid hydrogel was obtained by mixing equal volumes of PpIX–DNA and CD–DNA conjugate solutions in a heated NaCl buffer (Figure 23). Post-synthesis analyses consisted of in vitro PpIX release measurement, ROS generation from leached out PpIX assessment and antibacterial activity against *E. coli* et *S. aureus* evaluation.

The loading of PpIX within the polymeric matrix, either free or conjugated with DNA, can be confirmed by a change in fluorescence emission. The CD–DNA hydrogel caused an intense blue fluorescence when viewed under UV light, whereas PpIX–DNA, CD–DNA–PpIX and CD–DNA hydrogels (incubated with free PpIX for the latter) appeared red when observed under UV light. This feature helps to track the photosensitizer loading and release in the scope of PDT, which works as a therapy efficiency indicator. Regarding the ROS generation assessment, results showed a sustained release of PpIX over ten days through excitation under both UV (302 nm for 5 min) and visible light (300–900 nm for 5 min)—PpIX absorption in the UV range, where PpIX does not usually absorb, being possible thanks to a Forster resonance energy transfer (FRET) caused by CD. Basically, the energy absorbed by CD upon irradiation under UV light is ultimately transferred to PpIX. Nevertheless, ROS generation gradually decreased over time three times as less ROS were generated on the last day as compared to the first day. The conjugation of PpIX with DNA along with the CD–DNA crosslinking allowed the inhibition of the usual aggregation-induced PpIX self-quenching. Concerning bactericidal activity, the hybrid CD–DNA–PpIX hydrogel exhibited a significant antibacterial action after photoactivation by UV irradiation against *S. aureus* (302 nm), which was, however, dose-dependent. Once again, FRET between CD and PpIX enabled the PpIX excitation under UV irradiation and, thus, the generation of ROS. When irradiated under visible light (300–900 nm), over 99.99% of efficiency was observed in bacteria inactivation for the CD–DNA–PpIX hybrid hydrogel. The comparison of bacteria inactivation efficiency between the hydrogel and a solution of free PpIX tended to highlight the benefit of covalently immobilizing PpIX onto DNA to avoid PpIX self-quenching and hence increase the ROS generation efficiency. Their research group noticed a decreasing trend in killing efficacy over the days of the study, under exposure to both UV and visible light. No significant results were recovered concerning the killing efficacy of *E. coli* strains, which they explained by the membrane of Gram-negative bacteria being impermeable to neutral porphyrins. Overall, Kumari et al. designed a DNA, CD NPs and protoporphyrin IX hybrid polymeric network exhibiting tunable excitation energy-absorbing properties through FRET for ROS generation. The CD–DNA–PpIX hydrogel matrix was found to generate more ROS than free PpIX while avoiding aggregation-induced self-quenching of the photosensitizer through a sustained release of PpIX for enhanced aPDT applications.

## 4. Conclusions and Future Directions

Since their earliest investigation, the scientific interest in hydrogels and their applications has exponentially escalated over the years. Because they provide moisturizing environments, exhibit good biocompatibility, soft tissue-like viscoelastic properties, and excellent water retention capacity thanks to their three-dimensional porous network, hydrogels are particularly attractive candidates that have been extensively investigated as support matrices for biomedical applications. Antibacterial hydrogels either exhibit anti-fouling properties, cytotoxic activities or serve as antibacterial agent delivery systems, sometimes several of these characteristics at the same time. Their design is such that bactericidal effects can be the result of intrinsic antibacterial activities, the consequence of loaded antimicrobial agents slowly leaching into the environment or the outcome of an exterior factor such as light applied on the hydrogel surface.

However, the bactericidal action of antibacterial hydrogels tends to strongly decline over time, particularly for hydrogels loaded with antibacterial agents and inorganic nanoparticles, since their antimicrobial effect is often only effective upon the slow leaching of the bactericidal agent into the surrounding environment. Because of prompt leaching at the beginning of use and the rapid consumption of the antimicrobial agent, this phenomenon constitutes a major drawback for long-term antibacterial applications and raises environmental issues as toxic compounds can be released in various ecosystems. In the framework of long-lasting bactericidal effects and reusable/recyclable antibacterial hydrogels, the durability of each material and its antimicrobial effect duration must undergo systematic evaluation after several experimental bactericidal cycles.

The combination of antibacterial hydrogel and photochemistry came to light with photodynamic therapy. Upon irradiation under a specific wavelength, thanks to energy transfers between a chromophore-based photosensitizer in its excited state, which has been previously loaded into the hydrogel structure, and surrounding substrates and molecular oxygen, cytotoxic reactive oxygen species such as radicals and singlet oxygen are generated. Via denaturation of DNA, RNA and cellular proteins, reactive oxygen species induce apoptosis for surrounding bacteria. The study of photochemical processes further expanded in the scope of sustainable development and green chemistry with the investigation of photoinduced polymerization. Owing to the use of a photosensitive catalyst, the irradiation of a photocurable system at a given wavelength enables the crosslinking process to occur. Upon exposure to the appropriate light source, such a system is often solvent-free, offers quantitative results within minutes at ambient temperatures, allows spatial and temporal control, prevents the use of toxic substrates, the release of volatile organic compounds and harsh reactional environments while generating low levels of waste. It also usually calls for low costs of investment as photo-crosslinking can occur at low light intensities. The broad range of generic reactions which can be afforded by photoexcitation and the various kind of photopolymerizations available render the field of photochemistry all the more interesting in the framework of antibacterial hydrogels.

In future perspectives, the focus around photochemistry and antibacterial hydrogels should be put on the synthesis of new water-soluble photoinitiators, which would enable the use of eco-friendly and safe photocurable systems while avoiding the generation of phototoxic substances. The design of these photoinitiators would be such that they would be covalently incorporated within the final polymeric network in order to avoid their slow leaching out of the hydrogel structure and into the environment while ensuring the antibacterial hydrogel biocompatibility by preventing a local toxicity increase. Additionally, the use of photo-stable naturally occurring and easily modifiable dyes as synthons for the green synthesis of photosensitizers should further broaden the path to the generation of eco-friendly antibacterial hydrogels.

As briefly introduced in this review, the field of light-responsive antibacterial hydrogels is again widened by the concept of photothermal therapy (PTT). PTT enables the use of near-infrared light, a promising tool in the biomedical field as it can lead to novel materials with high antibacterial efficiencies thanks to deep tissue penetration, non-invasive treatment methods, low background scattering, low phototoxicity and accurate remote control. Future prospects on the union of light and antibacterial hydrogels may also lie on a hybrid approach between photodynamic and photothermal therapy; the antibacterial effects of the ROS generated by PDT combined with light converted into heat and thus inducing bacterial lysis through PTT is implied here. These techniques benefit from the use of visible light, non-invasive treatment methods, excellent availability of energy sources, spatial and temporal control, rapid results and mild environmental conditions. Hybrid materials containing dyes, photosensitizers and photothermal transduction entities benefit from the numerous advantages of these techniques described alongside this review.
